# Biomarkers associated with rhythm status after cardioversion in patients with atrial fibrillation

**DOI:** 10.1038/s41598-022-05769-9

**Published:** 2022-01-31

**Authors:** Pascal B. Meyre, Stefanie Aeschbacher, Steffen Blum, Gian Voellmin, Peter M. Kastner, Elisa Hennings, Beat A. Kaufmann, Michael Kühne, Stefan Osswald, David Conen

**Affiliations:** 1 Division of Cardiology, Department of Medicine, University Hospital Basel, Basel, Switzerland; 2 Cardiovascular Research Institute Basel, University Hospital Basel, Spitalstrasse 2, 4031 Basel, Switzerland; 3 Roche Diagnostics GmbH, Penzberg, Germany; 4 Population Health Research Institute, McMaster University, Hamilton, ON Canada

**Keywords:** Cardiology, Cardiovascular biology

## Abstract

Biomarkers may help to improve our knowledge about the complex pathophysiology of atrial fibrillation (AF). In this study we sought to identify significant changes in biomarkers and clinical measures in patients with and without AF recurrence after electrical cardioversion. We measured 21 conventional and new biomarkers before and 30 days after electrical cardioversion and assessed the associations of changes in biomarker levels with rhythm status at follow-up. Significant between-group changes were observed for bone morphogenetic protein 10 (BMP10), N-terminal pro-B-type natriuretic peptide (NT-proBNP) and total bilirubin. Their respective changes were − 10.4%, − 62.0% and − 25.6% in patients with sinus rhythm, and 3.1%, 1.1% and − 9.4% in patients with recurrent AF, for a between-group difference of − 13.5% (95% confidence interval [CI] − 19.3% to − 7.6%; P < 0.001), − 63.1% (95% CI − 76.6% to − 49.6%; P < 0.001) and − 16.3% (95% CI − 27.9% to − 4.7%; P = 0.007). In multivariable models, the reductions of BMP10 and NT-proBNP were significantly associated with follow-up rhythm status (β coefficient per 1 − SD decrease, − 3.85; 95% CI − 6.34 to − 1.35; P = 0.003 for BMP10 and − 5.84; 95% CI − 10.22 to − 1.47; P = 0.009 for NT-proBNP. In conclusion, changes in BMP10 und NT-proBNP levels were independently associated with rhythm status after cardioversion, suggesting that these markers may be dependent on the actual heart rhythm.

## Introduction

Although extensive epidemiological and experimental research has been conducted to identify risk factors for atrial fibrillation (AF), current knowledge of AF remains incomplete.

Identification of biomarkers related to AF may help to better understand the pathophysiological mechanisms of the arrythmia. Biomarkers of inflammation (C-reactive protein and fibrinogen)^1^, myocardial injury (high-sensitivity troponin I)^2^, renal dysfunction (fibroblast growth factor-23)^3^, and hemodynamic stress (NT-proBNP [N-terminal pro-B-type natriuretic peptide])^4–6^ have been reported to be independently associated with prevalent and incident AF. However, the observed relationships may at least in part be explained through well-established associations between biomarkers and cardiovascular conditions related to AF, as residual confounding is a potential issue for studies looking at between-group differences over a relatively long follow-up period. A within-person comparison during short-term follow-up may offer a more precise and unbiased picture of the associations between biomarker levels and rhythm status. Patients undergoing elective electrical cardioversion provide an ideal experimental framework for this purpose.

In the gene expression patterns for the prediction of atrial fibrillation (GAPP-AF) study we measured 11 conventional and new biomarkers, and performed laboratory measures in AF patients before and after electrical cardioversion. The objective of our study was to assess the association of changes in biomarkers with changes in heart rhythm status among patients with persistent AF undergoing elective electrical cardioversion.

## Results

### Baseline characteristics

Baseline characteristics stratified by rhythm status at follow-up are presented in Table 1. During follow-up, 28 (28%) patients had a documented AF recurrence. There were no statistically significant between-group differences. During 30-day follow-up no significant change in medication was observed in both groups (Table S1).

**Table 1:** Baseline characteristics of patients with and without AF recurrence after cardioversion.

Characteristic	All patients (N = 100)	Patients with AF recurrence (N = 28)	Patients in sinus rhythm (N = 72)	P value
Age, years	66 ± 10	67 ± 8	66 ± 11	0.61
**Sex, no. (%)**				0.44
Women	25 (25)	5 (18)	20 (28)	
Men	75 (75)	23 (82)	52 (72)	
Body-mass index, median (IQR), kg/m^2^	26.0 (24.2–29.5)	25.7 (22.9–29.5)	27.0 (24.4–29.5)	0.63
**Smoking status, no. (%)**				0.07
Active	11 (11)	2 (7)	9 (12)	
Past	49 (49)	19 (68)	30 (42)	
Never	40 (40)	7 (25)	33 (46)	
Months since atrial fibrillation diagnosis, median (IQR)	16 (4–76)	9 (4–47)	19 (4–79)	0.31
**Medical history, no. (%)**				
Hypertension	64 (64)	21 (75)	43 (60)	0.17
Diabetes mellitus	6 (6)	2 (7)	4 (6)	0.67
Stroke or transient ischemic attack	11 (11)	2 (7)	9 (13)	0.72
Myocardial infarction	8 (8)	2 (7)	6 (8)	0.99
Percutaneous coronary intervention	12 (12)	3 (11)	9 (13)	0.99
Heart failure	14 (14)	5 (18)	9 (13)	0.53
**Medication, no. (%)**				
Beta-blocker	76 (76)	18 (64)	58 (82)	0.11
Calcium channel blockers	15 (15)	5 (18)	10 (14)	0.76
Flecainide	4 (4)	1 (4)	3 (4)	0.99
Amiodarone	41 (41)	10 (36)	31 (44)	0.51
Dronedarone	7 (7)	3 (11)	4 (6)	0.40
Digoxin or digitoxin	8 (8)	3 (11)	5 (7)	0.68

Values are means ± SD or median (IQR) and numbers (percent).

P values compare patients between with AF recurrence and sinus rhythm and are obtained from Wilcoxon rank-sum tests for continuous variables and Fisher’s exact tests for categorical variables.

### Changes in biomarkers by rhythm status at follow-up

**Figure 1:**
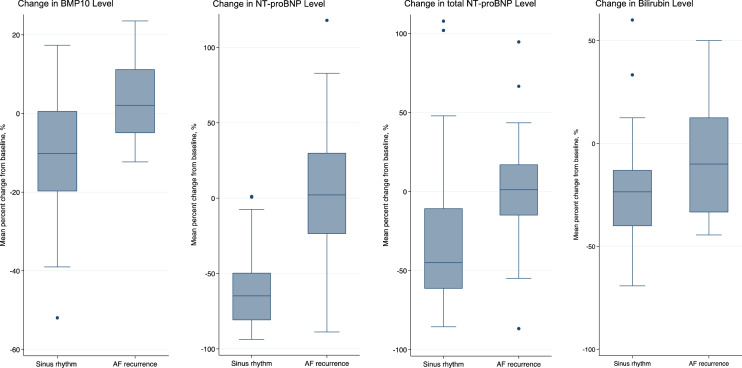
Box plots of most significant percent changes in biomarker levels by rhythm status after cardioversion. Y-axis represents mean percent change in biomarker level; horizontal bars are median values; boxes, 25th and 75th percentiles; error bars, interquartile ranges; and dots, outliers.

**Figure 2:**
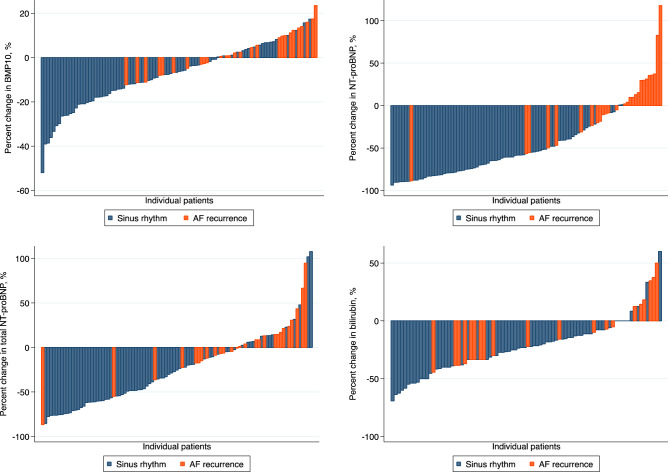
Waterfall plots of percent changes in biomarker levels by rhythm status after cardioversion. X-axis represents individual patients, y-axis percentage change; bars are percent change of biomarker at follow-up for each patient.

Absolute values of biomarker levels and clinical measures at baseline and follow-up are presented in Table 2 and Table S2. Differences in biomarker levels according to rhythm status at follow-up are shown in Table 3 and Fig. S1. Between-group differences exceeding the threshold of P < 0.01 were observed for BMP10, NT-proBNP, total NT-proBNP and total bilirubin (Fig. 1). Waterfall plots of individual percent change in biomarker levels are presented in (Fig. 2). There were significant correlations between levels of BMP10, NT-proBNP, total NT-proBNP, and BNP at baseline and follow-up, but not for total bilirubin, as shown in Tables S3, S4, Figs. S2 and S3.

**Table 2:** Absolute biomarker levels at baseline and follow-up stratified by rhythm status at follow-up.

Biomarkers	Baseline			Follow-up		
	AF recurrence	Sinus rhythm	P value	AF recurrence	Sinus rhythm	P value
FABP3, ng/mL	37.26 ± 11.36	37.82 ± 15.63	0.43	34.74 ± 12.91	34.08 ± 14.04	0.68
ESM1, ng/mL	2.39 ± 0.82	2.44 ± 0.98	0.99	2.34 ± 0.74	2.15 ± 0.68	0.13
BMP10, ng/mL	2.38 ± 0.56	2.40 ± 0.72	0.79	2.46 ± 0.65	2.09 ± 0.48	0.0076
DKK3, ng/mL	62.67 ± 18.71	62.82 ± 16.87	0.84	64.16 ± 21.21	59.91 ± 16.92	0.50
FGF23, ng/mL	268.27 ± 259.71	338.05 ± 726.94	0.13	267.50 ± 215.78	244.68 ± 469.87	0.0363
IGFBP7, ng/mL	108.77 ± 34.87	103.17 ± 25.75	0.67	110.71 ± 28.98	101.30 ± 22.72	0.16
MyBPC3, ng/L	19.15 ± 13.50	21.61 ± 34.44	0.45	23.01 ± 19.18	21.71 ± 22.33	0.57
NT-proBNP, pg/mL	1167.39 ± 690.88	1311.35 ± 1156.85	0.99	1165.03 ± 887.36	512.82 ± 522.43	< 0.001
total NT-proBNP, pg/mL	3672.49 ± 2668.65	3822.49 ± 2916.65	0.99	3702.79 ± 2765.81	2455.07 ± 1931.88	0.0200
BNP, pg/mL	488.71 ± 751.64	336.60 ± 265.16	0.99	302.76 ± 195.05	224.25 ± 203.08	0.0147
hsTnT, pg/mL	13.78 ± 11.45	11.97 ± 11.05	0.39	15.67 ± 12.97	11.69 ± 9.69	0.16
Sodium, mmol/L	140.52 ± 2.36	140.68 ± 2.03	0.80	141.13 ± 2.47	140.96 ± 2.21	0.81
Potassium, mmol/L	4.18 ± 0.40	4.11 ± 0.46	0.24	4.23 ± 0.29	4.16 ± 0.43	0.46
Creatinine, umol/L	89.69 ± 27.03	87.09 ± 22.28	0.98	91.42 ± 28.68	89.12 ± 31.14	0.89
Urea, mmol/L	7.27 ± 3.01	6.77 ± 2.42	0.65	7.16 ± 1.90	7.51 ± 3.69	0.99
Total bilirubin, umol/L	11.88 ± 6.12	13.46 ± 5.39	0.11	10.50 ± 6.52	9.62 ± 4.25	0.86
Aspartate aminotransferase, U/L	30.46 ± 8.66	31.32 ± 9.66	0.79	32.13 ± 10.17	31.86 ± 9.37	0.86
Alanine aminotransferase, U/L	29.75 ± 15.62	34.25 ± 16.70	0.18	28.25 ± 13.01	33.25 ± 16.25	0.25
C-reactive protein, mg/L	2.11 ± 2.85	4.14 ± 8.91	0.24	2.64 ± 4.11	2.95 ± 3.55	0.38
Lactate dehydrogenase, U/L	230.87 ± 63.95	226.43 ± 64.28	0.73	246.46 ± 67.84	218.86 ± 51.49	0.14
Creatine kinase, U/L	140.96 ± 89.65	109.56 ± 94.38	0.0115	134.71 ± 68.23	131.40 ± 95.38	0.32

*FABP3* fatty acid binding protein 3, *ESM1* endothelial cell-specific molecule-1, *BMP10* bone morphogenetic protein 10, *DKK3*, dickkopf related protein-3, *FGF23* fibroblast growth factor 23, *IGFBP7* insulin growth factor binding protein-7, *MyBPC3* myosin-binding protein C, *NT-proBNP* N-terminal pro-B-type natriuretic peptide, *BNP* brain natriuretic peptide, *hsTnT* high-sensitive troponin T.

Values are mean ± standard deviation.

P value compares biomarkers levels between patient with AF recurrence and patients with sinus rhythm and are calculated using Wilcoxon rank-sum test.

**Table 3:** Changes in biomarker concentrations by rhythm status at follow-up.

Biomarkers	Absolute change in markers (95% CI)^a^			Percent change in markers (95% CI)			P value
	AF recurrence	Sinus rhythm	Between-Group Difference	AF recurrence	Sinus rhythm	Between-Group Difference	
FABP3, ng/mL	− 2.34 (− 5.52 to 0.84)	− 3.75 (− 5.71 to − 1.80)	− 1.41 (− 5.10 to 2.28)	− 5.95 (− 14.33 to 2.43)	− 8.43 (− 12.54 to − 4.31)	− 2.48 (− 10.79 to 5.84)	0.45
ESM1, ng/mL	− 0.03 (− 0.19 to 0.12)	− 0.29 (− 0.45 to − 0.13)	0.26 (− 0.54 to 0.03)	0.59 (− 6.05 to 7.24)	− 8.13 (− 12.29 to − 3.98)	− 8.73 (− 16.54 to − 0.91)	0.07
BMP10, ng/mL	0.07 (− 0.03 to 0.19)	− 0.31 (− 0.42 to − 0.19)	− 0.38 (− 0.57 to − 0.19)	3.07 (− 0.78 to 6.92)	− 10.40 (− 13.70 to − 7.10)	− 13.47 (− 19.31 to − 7.63)	< 0.001
DKK3, ng/mL	1.69 (− 1.80 to 5.18)	− 2.91 (− 4.97 to − 0.86)	− 4.61 (− 8.52 to − 0.69)	3.02 (− 2.06 to 8.09)	− 3.74 (− 7.19 to − 0.29)	− 6.76 (− 13.10 to − 0.41)	0.0216
FGF23, ng/mL	− 1.43 (− 37.92 to 35.06)	− 93.38 (− 232.31 to 45.55)	− 91.95 (− 319.50 to 135.61)	6.34 (− 6.23 to 18.90)	− 7.12 (− 13.84 to − 0.39)	− 13.45 (− 26.66 to − 0.24)	0.42
IGFBP7, ng/mL	2.04 (− 3.32 to 7.39)	− 1.88 (− 5.45 to 1.70)	− 3.91 (− 10.53 to 2.71)	3.31 (− 0.36 to 6.99)	− 0.42 (− 3.47 to 2.64)	− 3.73 (− 9.16 to 1.70)	0.24
MyBPC3, ng/L	4.13 (− 0.44 to 8.70)	0.31 (− 5.04 to 5.70)	− 3.80 (− 12.91 to 5.30)	24.31 (1.19 to 47.44)	20.02 (11.20 to 28.83)	− 4.30 (− 24.05 to 15.45)	0.41
NT-proBNP, pg/mL	5.1 (− 196.8 to 207.0)	− 798.5 (− 986.4 to − 610.7)	− 803.6 (− 1132.0 to − 475.2)	1.09 (− 16.18 to 18.35)	− 62.02 (− 67.54 to − 56.50)	− 63.11 (− 76.64 to − 49.57)	< 0.001
total NT-proBNP, pg/mL	− 7.8 (− 514.5 to 530.1)	− 1367.4 (− 1821.2 to − 913.6)	− 1375.2 (− 2176.0 to − 574.4)	2.66 (− 11.15 to 16.46)	− 33.35 (− 42.52 to − 24.19)	− 36.01 (− 53.01 to − 19.01)	0.001
BNP, pg/mL	− 19.40 (− 110.31 to 71.52)	− 120.98 (− 171.02 to − 70.93)	− 101.58 (− 200.03 to − 3.14)	10.70 (− 6.99 to 28.38)	− 30.65 (− 43.65 to − 17.65)	− 41.34 (− 65.35 to − 17.34)	0.0433
hsTnT, pg/mL	1.50 (0.14 to 2.85)	− 0.32 (− 1.71 to 1.08)	− 1.81 (− 4.21 to 0.58)	6.87 (− 10.98 to 24.71)	5.16 (− 6.66 to 16.97)	− 1.71 (− 23.56 to 20.13)	0.11
Sodium, mmol/L	0.33 (− 0.87 to 1.54)	0.16 (− 0.46 to 0.79)	− 0.17 (− 1.41 to 1.07)	0.26 (− 0.60 to 1.11)	0.13 (− 0.31 to 0.58)	− 0.12 (− 1.01 to 0.76)	0.79
Potassium, mmol/L	0.05 (− 0.10 to 0.19)	0.05 (− 0.04 to 0.15)	0.008 (− 0.17 to 0.18)	1.69 (− 1.92 to 5.30)	1.82 (− 0.40 to 4.05)	0.14 (− 4.10 to 4.38)	0.93
Creatinine, umol/L	1.39 (− 2.43 to 5.22)	− 0.09 (− 4.32 to 4.14)	− 1.48 (− 8.91 to 5.94)	1.58 (− 2.41 to 5.57)	− 0.32 (− 4.29 to 3.64)	− 1.90 (− 8.93 to 5.12)	0.69
Urea, mmol/L	− 0.13 (− 0.85 to 0.58)	0.65 (0.14 to 1.17)	0.79 (− 0.17 to 1.74)	3.02 (− 4.73 to 10.76)	9.95 (3.75 to 15.52)	6.62 (− 4.13 to 17.37)	0.10
Total bilirubin, umol/L	− 1.39 (− 3.03 to 0.25)	− 3.75 (− 4.59 to − 2.91)	− 2.35 (− 4.03 to − 0.68)	− 9.35 (− 21.14 to 2.44)	− 25.64 (− 31.37 to − 19.91)	− 16.29 (− 27.92 to − 4.66)	0.0065
Aspartate aminotransferase, U/L	2.13 (0.45 to 3.81)	0.76 (− 1.18 to 2.70)	− 1.37 (− 4.72 to 1.98)	7.06 (1.53 to 12.59)	5.91 (− 0.28 to 12.09)	− 1.15 (− 11.86 to 9.55)	0.42
Alanine aminotransferase, U/L	0.30 (− 2.13 to 2.74)	− 0.84 (− 4.81 to 3.13)	− 1.15 (− 7.86 to 5.57)	3.70 (− 7.40 to 14.80)	8.24 (− 5.06 to 21.55)	4.55 (− 18.35 to 27.44)	0.73
C-reactive protein, mg/L	0.37 (− 1.15 to 1.89)	− 1.26 (− 3.26 to 0.73)	− 1.63 (− 5.05 to 1.78)	20.23 (− 7.28 to 47.73)	25.97 (0.44 to 51.50)	5.74 (− 39.29 to 50.78)	0.34
Lactate dehydrogenase, U/L	17.26 (0.83 to 33.69)	− 9.63 (− 22.80 to 3.53)	− 26.90 (− 50.61 to − 3.18)	8.39 (1.41 to 15.36)	− 1.53 (− 6.00 to 2.93)	− 9.92 (− 18.31 to − 1.52)	0.0267
Creatine kinase, U/L	− 4.39 (− 22.16 to 13.38)	19.11 (6.63 to 31.59)	23.50 (0.38 to 46.62)	3.19 (− 10.01 to 16.39)	21.92 (11.01 to 32.83)	18.73 (− 0.93 to 38.39)	0.0465

*FABP3* fatty acid binding protein 3, *ESM1* endothelial cell-specific molecule-1, *BMP10* bone morphogenetic protein 10, *DKK3*, dickkopf related protein-3, *FGF23* fibroblast growth factor 23, *IGFBP7* insulin growth factor binding protein-7, *MyBPC3* myosin-binding protein C, *NT-proBNP* N-terminal pro-B-type natriuretic peptide, *BNP* brain natriuretic peptide, *hsTnT* high-sensitive troponin T.

^a^Mean difference with corresponding 95% confidence intervals.

P value compares mean differences of biomarkers between patient with AF recurrence and patients with sinus rhythm and are calculated using 2-sample t tests.

### Changes in clinical measures by rhythm status at follow-up

Changes in clinical measures are presented in Table 4. We observed significant between-group differences in average and maximum heart rate (between-group difference, − 17.3%; 95% CI − 30.0 to − 4.6 and − 29.2%; 95% CI − 41.2 to − 17.3, respectively). Because NT-proBNP and total NT-proBNP were highly correlated (Spearman’s rho at baseline and follow-up, 0.9364 and 0.918 (Tables S3, S4)), we used only NT-proBNP for further investigation given its routine availability in clinical practice.

**Table 4:** Changes in clinical measures by rhythm status at follow-up.

	Absolute change in markers (95% CI)^a^			Percent change in markers (95% CI)			P value
	AF recurrence	Sinus rhythm	Between-group difference	AF recurrence	Sinus rhythm	Between-group difference	
**Blood pressure measures**							
Systolic, mmHg	− 5.17 (− 13.14 to 2.79)	− 1.49 (− 5.66 to 2.68)	3.68 (− 4.66 to 12.02)	− 2.96 (− 8.14 to 2.22)	− 0.53 (− 3.47 to 2.40)	2.43 (− 3.32 to 8.18)	0.38
Diastolic, mmHg	− 1.85 (− 7.43 to 3.73)	− 9.29 (− 12.41 to − 6.16)	− 7.44 (− 13.58 to − 1.30)	− 1.20 (− 6.95 to 4.55)	− 9.30 (− 12.75 to − 5.85)	− 8.10 (− 14.77 to − 1.44)	0.0181
**Holter ECG parameters**							
Average heart rate, /min	− 6.50 (− 16.22 to 3.22)	− 22.13 (− 26.44 to − 17.81)	− 15.63 (− 24.80 to − 6.45)	− 6.57 (− 19.27 to 6.13)	− 23.89 (− 30.01 to − 17.76)	− 17.31 (− 30.00 to − 4.63)	0.0011
Max. heart rate, /min	5.29 (− 11.00 to 21.58)	− 41.56 (− 50.89 to − 32.22)	− 46.84 (− 65.26 to − 28.42)	5.92 (− 6.47 to 18.31)	− 23.31 (− 29.01 to − 17.61)	− 29.23 (− 41.18 to − 17.28)	< 0.001
Min. heart rate, /min	1.10 (− 2.83 to 5.02)	− 3.44 (− 6.26 to − 0.63)	− 4.54 (− 9.86 to 0.78)	4.76 (− 3.50 to 13.03)	− 4.03 (− 10.55 to 2.50)	− 8.79 (− 20.95 to 3.37)	0.09

^a^Mean difference with corresponding 95% confidence intervals.

P value compares mean differences of biomarkers between AF recurrence and sinus rhythm patients and are calculated using 2-sample t tests.

### Associations of change in biomarker levels with rhythm status after cardioversion

Changes in significant biomarker levels (BMP10 and NT-proBNP) remained associated with rhythm status when logistic models were separately adjusted for significant changes in clinical measures (Table S5). In models adjusted for age and sex, change in BMP10 was significantly associated with rhythm status at follow-up (standardized β, − 2.03; 95% CI − 3.06 to − 1.00; P < 0.001), shown in Table 5. Results remained similar with additional adjustment for demographic characteristics, risk factors for AF and changes in clinical measures. Similarly, there was a strong association between change in NT-proBNP and rhythm status at follow-up after age and sex adjustment (standardized β, − 3.98; 95% CI − 5.75 to − 2.22; P < 0.001) and this relationship remained significantly associated after additional multivariable adjustments. Change in total bilirubin was not significantly associated with rhythm status at follow-up after extensive multivariable adjustment, as shown in Table 5.

**Table 5:** Associations of change in biomarker levels with rhythm status after cardioversion.

Model	Predictor								
	Change in BMP10, ng/mL			Change in NT-proBNP, pg/mL			Change in total bilirubin, umol/L		
	β (95% CI)	Standardized β (95% CI)^c^	P value	β (95% CI)	Standardized β (95% CI)	P value	β (95% CI)	Standardized β (95% CI)	P value
Univariable	− 4.25 (− 6.40 to − 2.10)	− 1.97 (− 2.96 to − 0.97)	< 0.001	− 0.005 (− 0.007 to − 0.003)	− 3.93 (− 5.63 to − 2.23)	< 0.001	− 0.24 (− 0.43 to − 0.04)	− 0.85 (− 1.56 to − 0.15)	0.018
Adjusted for age and sex	− 4.39 (− 6.62 to − 2.15)	− 2.03 (− 3.06 to − 1.00)	< 0.001	− 0.005 (− 0.007 to − 0.003)	− 3.98 (− 5.75 to − 2.22)	< 0.001	− 0.26 (− 0.48 to − 0.04)	− 0.95 (− 1.71 to − 0.20)	0.014
Multivariable model 1^a^	− 5.30 (− 8.08 to − 2.53)	− 2.47 (− 3.76 to − 1.18)	< 0.001	− 0.006 (− 0.009 to − 0.003)	− 4.67 (− 6.90 to − 2.43)	< 0.001	− 0.26 (− 0.49 to − 0.03)	− 0.92 (− 1.74 to − 0.09)	0.029
Multivariable model 2^b^	− 8.08 (− 13.32 to − 2.83)	− 3.85 (− 6.34 to − 1.35)	0.003	− 0.01 (− 0.02 to − 0.002)	− 5.84 (− 10.22 to − 1.47)	0.009	− 0.36 (− 0.75 to 0.04)	− 1.32 (− 2.77 to 0.13)	0.08

Estimates were obtained from logistic regression models where rhythm status at follow-up was the dependent variable and change of biomarker was the independent variable. All models were adjusted for biomarker levels at baseline.

^a^Adjusted for age (years), sex, BMI (kg/m^2^), smoking status (active, past, never), hypertension (yes vs no), heart failure (yes vs no).

^b^Multivariable model 1, plus change in clinical parameters (diastolic BP, average heart rate, max. heart rate).

^c^Standardized β coefficients are defined as per 1-SD decrease in biomarker change.

None of the patients who had a reduction of both BMP10 and NT-proBNP levels below the respective median had an AF recurrence during follow-up (Table S6).

## Discussion

In this prospective study of patients undergoing electrical cardioversion for persistent AF, patients who were in sinus rhythm after 30 days of follow-up had considerable reductions in BMP10 and NT-proBNP levels, whereas patients with AF recurrence had no significant change in these biomarker levels. The reductions of BMP10 and NT-proBNP remained significantly associated with rhythm status at follow-up after multivariable adjustment, suggesting that these biomarker changes may reflect the change in rhythm status itself, given that most other covariates and comorbidities remained unchanged during the short follow-up of 30 days. In contrast, adjusting for change in average heart rate did not have an impact on these associations, suggesting no association between these biomarker changes with actual heart rate.

Circulating blood biomarkers have the potential to provide insights into the pathogenesis of AF and enhance risk prediction. We looked at a large set of 21 biomarkers and BMP10 and natriuretic peptides (including NT-proBNP and total NT-proBNP) were among the most significant ones. BMP10 is a heart-restricted protein that plays a role in cardiomyocyte development and growth^7^. Genes encoding for BMP10 are strongly expressed in the atria^8^. The secretion of BMP10 is regulated by *PITX2*, a gene variant known to be associated with AF^9–11^. Reduced expression of *PITX2* results in both higher levels of BMP10 and higher risk for development of AF^9^. BMP10 mRNA is expressed at low to undetectable levels in left ventricular tissue^9^, and appears not to be affected by other cardiovascular conditions that associate with AF such as heart failure^12^. A recently published study performed in patients who underwent AF ablation found a strong decrease in BMP10 levels among those patients who were in sinus rhythm at 3 months of follow-up, whereas BMP10 levels remained unchanged among those who had AF recurrence^13^. Our study not only confirms these latter findings, but also shows that BMP10 may be directly linked to the actual heart rhythm. BMP10 levels did not differ between groups prior to cardioversion (Table 2), but only when one group achieved stable sinus rhythm at 30 days. Patients who remained in AF had persistent increased BMP10 levels, and only those who converted into a stable sinus rhythm had significant reductions in BMP10 levels.

The underlying biologic mechanisms for increased BMP10 levels in AF compared with sinus rhythm are unclear. Given that BMP10 plays a role in myocardial cell growth, overexpression of BMP10 may induce an excess in myocardial remodeling and facilitate the persistence and/or recurrence of AF. Furthermore, it has been suggested that BMP10 may have inflammatory properties that may induce AF development^14^. Given that BMP10 levels are closely related to the heart rhythm, future studies should investigate whether BMP10 alone or in addition to well known risk factors for AF has the potential to improve AF screening.

Natriuretic peptides including BNP and NT-proBNP have been well described as biomarkers associated with AF^4,15–17^. However, there are differences in analytical characteristics, pathophysiological interpretations, and clinical relevance between the measurement of BNP and NT-proBNP^18^. It has been shown that the ratio of NT-proBNP/BNP is not a constant value but varies according to various diseases and conditions^19^. Also, a great part of the differences between the various BNP immunoassays seems to be influenced by the cross-reaction with the glycosylated or not glycosylated proBNP^20^. In the absence of heart failure, it has been demonstrated that NT-proBNP is released primarily by the atria because of the increased myocardial contraction, volume load and wall stress caused by AF^21^. Restoration of sinus rhythm through cardioversion has been shown to reduce natriuretic peptides concentrations^22–24^. Our data extend these previous reports by demonstrating within-person reductions in NT-proBNP during a short-term follow-up in patients who were in sinus rhythm, and persistently elevated levels in those who remained in AF. This reduction was independently associated with sinus rhythm at follow-up, even after adjusting for clinical variable changes including heart rate, suggesting that changes in NT-proBNP are at least in part related to the heart rhythm. The potential mechanism could be that restoration of sinus rhythm reduces myocardial contraction and atrial wall stress which inhibits NT-proBNP secretion.

Previous studies have shown that elevated levels of FGF23 are associated with AF^3,25^. We observed small reductions in FGF23 levels in patients who were in sinus rhythm at follow-up, but it was not statistically significant. However, FGF23 has been reported to play a role in myocardial remodeling and endothelial dysfunction^26,27^, lending support to the concept that FGF23 may be a marker of atrial cardiomyopathy^28^.

Taken together, the findings of our study show that both BMP10 and NT-proBNP seem strongly dependent on the actual heart rhythm, suggesting that using these biomarkers may help to identify patients with undiagnosed AF. Although opportunistic screening for AF either by ECG monitoring or pulse taking in patients older than 65 years is recommended in guidelines^29^, studies indicate that such a screening strategy alone may not increase the detection rate of AF^30,31^. Incorporating biomarkers into established screening strategies may therefore improve AF detection rates^32,33^. A prospective cohort study found that a NT-proBNP-stratified systematic screening for AF identifies more AF cases in high-risk individuals^34^. Our data provide evidence that not only NT-proBNP but also BMP10 are dependent on rhythm status suggesting that both biomarkers may be useful to detect AF. Future studies should assess whether biomarker testing using BMP10 and NT-proBNP has the potential to enhance identification of patients with undiagnosed AF.

We observed a significant reduction in total bilirubin in patients who were in sinus rhythm after cardioversion. However, this reduction did not remain statistically significant in multivariable analyses. Total bilirubin may therefore reflect an increased central venous pressure and mild liver congestion occurring in AF, rather than a direct effect of AF itself. Nevertheless, increased oxidative stress occurring during AF may further explain this association^35^, given the antioxidant properties of bilirubin^36,37^.

Although no significant difference in hs-TnT levels was found in patients who were in sinus rhythm after cardioversion compared to those who had AF recurrence, we observed elevated hs-TnT levels above the 99th percentile of the upper reference limit (> 14 pg/mL) in 28 patients at both baseline and follow-up (Table S7), suggesting that a significant proportion of these patients have some form of myocardial damage and/or increased troponin leakage. Prior studies have reported elevated hs-TnT levels in patients with AF^38–40^. The underlying mechanisms are probably multifactorial and may include myocardial distress and increased oxygen demand (i.e. due to increased or irregular heart rates), myocardial dysfunction caused by variations in atrial and ventricular volume and pressure load, and the presence of comorbidities associated with myocardial damage such as heart failure and history of myocardial infarction. Given the high correspondence of elevated hs-TnT levels in our study, it is very likely that these values reflect chronic elevations and are not related to the rhythm status.

The key strength of this prospective study is that we used a quasi-experimental setting to assess changes in a large number of conventional and new biomarker levels during AF and in sinus rhythm after cardioversion. However, there are potential limitations that deserve discussion. First, some patients with AF recurrence refused to attend the follow-up visit and follow-up blood samples were unavailable in these patients. Second, we included a relatively small sample of patients with persistent AF undergoing electrical cardioversion, and the generalizability to other AF populations remains unclear. Due to the small sample size of our study, overadjustment of the models may have been an issue. However, the confidence intervals of the estimates were stable after including additional covariates making overadjustment unlikely. Third, we did not include transthoracic echocardiographic imaging measures in the analyses because of (1) missing data and (2) of suboptimal visualization of the atrial and ventricular cavities due to the irregular contraction in AF^41^. In addition, adjusting for echocardiographic variables may over-adjust the observed relationships by adjusting for variables in the causal pathway. Women were underrepresented in our study. Forth, follow-up screening for AF did not include continuous rhythm monitoring. It is possible that some intermittent AF recurrences may have been missed. However, all patients had a 24-h ECG recording to document the actual rhythm status immediately prior to the follow-up visit where follow-up blood samples were drawn. Actual rhythm status was therefore known in all patients for 24 h, such that it is highly unlikely that missed AF episodes prior to that had a substantial effect on the observed biomarker levels, and thereby on the relationship between the change in biomarker level and rhythm status. Finally, given that our study was hypothesis-generating, future studies are needed to validate our findings in other larger cohorts of patients without diagnosed AF.

In this prospective study, significant reductions of BMP10 und NT-proBNP levels were observed in patients who converted to sinus rhythm after cardioversion, but not in those with AF recurrence. Our analyses showed that even after multivariable adjustments, the results remained consistent, suggesting that these markers may be related, at least in part, to rhythm status in AF. The role of BMP10 in the identification of patients with undiagnosed AF should be examined in future studies.

## Methods

### Study design and population

All study participants were from the GAPP-AF study, a prospective study of AF patients who were scheduled for a non-urgent electrical cardioversion. Eligibility criteria were age ≥ 18 years and patients had to have persistent AF, defined as a non-self terminating AF episode lasting > 7 days. In addition, patients had to be on appropriate oral anticoagulation for at least 3 weeks prior to cardioversion. Exclusion criteria were acute heart failure, severe valvular disease, life-limiting active or chronic major diseases, and a history of cardiac surgery within 3 months prior to enrollment. Of the 128 patients enrolled into the study (enrollment period from March, 2010 to April, 2013), 28 were excluded from the analysis (Fig. S4). The main reason was that patients did not attend the follow-up visit (n = 24). An additional 4 patients had missing information on biomarker levels at baseline, thus leaving a total of 100 patient for the present analysis. The study was conducted in accordance with the Declaration of Helsinki and was approved by the local ethics committee, Ethikkommission Nordwest- und Zentralschweiz (EKNZ). Written informed consent was obtained from all participants.

### Baseline study procedures

After providing informed consent, all patients had a 24-h Holter electrocardiogram (ECG) prior to the cardioversion to confirm the presence of persistent AF, and to document the heart rate profile. Baseline characteristics and comorbidities were collected using standardized case report forms. Three consecutive blood pressure measurements were obtained, and the mean of them was used in all analyses. Immediately before cardioversion, non-fasting venous blood samples were drawn for biomarker analyses. Electrical cardioversion was performed according to institutional guidelines.

### Follow-up and outcome

For all patients, a follow-up visit was scheduled 30 days after cardioversion. Patients were advised to obtain an ECG every time they experience palpitations or other symptoms of potential AF recurrence. Immediately before the follow-up visit, all patients had another 24-h ECG recording to document the prevalent rhythm. During the visit, three consecutive blood pressure measurements were obtained, and follow-up blood samples were drawn. Recurrence of AF was defined as any AF episode lasting longer than 30 s on 24-h ECG monitoring^42^, or any ECG recording showing AF obtained during the follow-up period.

### Biomarker testing

Immediately after the blood draw, plasma samples were centrifuged, aliquoted and stored at − 80 °C in a central biobank. Biomarkers were analyzed by personnel blinded to rhythm status. New candidate biomarkers were evaluated based on their associations with cardiovascular disease; myocardial injury (FABP3), endothelial remodeling (ESM1), atrial tissue (BMP10), chronic kidney disease (DKK3), cardiac hypertrophy (IGFBP7, MyBPC3), and elevated left ventricular filling pressure (total NT-proBNP). Levels of BMP10 were measured from EDTA plasma using a research grade prototype assay on a Cobas Elecsys e601 platform (Roche Diagnostics GmbH, Mannheim) employing Elecsys electrochemiluminescence technology. N-terminal pro-B-type natriuretic peptide (NT-proBNP) and total NT-proBNP levels were measured using a commercial sandwich immunoassay (NT-proBNP II, Roche Diagnostics GmbH, Mannheim) and a research grade prototype assay on the Elecsys e601 platform. Other biomarkers that were measured included: Fatty acid-binding protein 3, endothelial cell-specific molecule 1, dickkopf-related protein 3, insulin-like growth factor-binding protein 7, myosin-binding protein C 3, high-sensitivity cardiac troponin T. More details on these biomarkers are provided in the Supplement. In addition, a number of laboratory measures were obtained from fresh blood samples that were immediately analyzed in the same hospital laboratory at baseline and during follow-up.

### Statistical analysis

Continuous variables are presented as mean ± standard deviation (SD) or median (interquartile range, [IQR]), as appropriate. Categorical data are presented as number (percentage). The distribution of continuous variables was checked using kurtosis and skewness, and by visual inspection of the histogram. Baseline variables were stratified by rhythm status at follow-up and compared using Wilcoxon rank-sum tests or Fisher’s exact tests, as appropriate. Changes from baseline to follow-up in biomarker levels and clinical measures were compared between patients with and without AF recurrence during follow-up. Because change values did not show a skewed distribution, we used 2-sample t-tests to compare changes in biomarkers and clinical measures. Variables were retained for further analysis if the P for difference reached the predefined threshold of < 0.01. Correlations between significant biomarkers were evaluated at baseline and follow-up using Spearman’s correlation coefficients and combined scatter and linear prediction plots were used to quantify these correlations. Differences between patients with and without AF recurrence were further investigated using logistic regression models with rhythm status as the dependent variable and change in biomarker levels or clinical variables as the independent variable. Separate models were fitted for each individual variable reaching the predefined significance threshold of 0.01. Baseline biomarker value was included as a covariate in each model. In order to compare coefficients between biomarkers, we calculated β coefficients per change in 1 standard deviation (SD). Multivariable models were then adjusted for age, sex, smoking status, hypertension, heart failure (model 1), and model 1 plus significant changes in clinical measures, including diastolic BP, average heart rate and maximal heart rate (model 2). To evaluate whether the association between changes in the most significant biomarkers and rhythm status provides incremental information, we created 4 mutually exclusive groups, according to whether the change of biomarker levels was below or above the respective median of biomarker change and multivariable adjusted models were used to compare groups.

All statistical analyses were performed using STATA, version 17.0 (StataCorp LLC). Data analysis was performed from September 8, 2020, to November 20, 2020 and no additional patients were included between the end of the enrollment period and statistical analyses.

## Supplementary Information

Supplementary Information.

## Abbreviations

AF: Atrial fibrillation
BMP10: Bone morphogenetic protein 10
BNP: Brain natriuretic peptide
ECG: Electrocardiogram
GAPP-AF: Gene expression patterns for the prediction of atrial fibrillation
mRNA: Messenger ribonucleic acid
NT-proBNP: N-terminal pro-B-type natriuretic peptide

## References

[1] Conen D (2010). A multimarker approach to assess the influence of inflammation on the incidence of atrial fibrillation in women. Eur. Heart J..

[2] Rienstra M (2014). Relation between soluble ST2, growth differentiation factor-15, and high-sensitivity troponin I and incident atrial fibrillation. Am. Heart J..

[3] Chua W (2019). Data-driven discovery and validation of circulating blood-based biomarkers associated with prevalent atrial fibrillation. Eur. Heart J..

[4] Schnabel RB (2010). Relations of biomarkers of distinct pathophysiological pathways and atrial fibrillation incidence in the community. Circulation.

[5] Ellinor PT, Low AF, Patton KK, Shea MA, Macrae CA (2005). Discordant atrial natriuretic peptide and brain natriuretic peptide levels in lone atrial fibrillation. J. Am. Coll. Cardiol..

[6] Staerk L (2020). Protein biomarkers and risk of atrial fibrillation: The FHS. Circ. Arrhythm Electrophysiol..

[7] Chen H (2004). BMP10 is essential for maintaining cardiac growth during murine cardiogenesis. Development.

[8] Hsu J (2012). Whole genome expression differences in human left and right atria ascertained by RNA sequencing. Circ. Cardiovasc. Genet..

[9] Reyat JS (2020). Reduced left atrial cardiomyocyte PITX2 and elevated circulating BMP10 predict atrial fibrillation after ablation. JCI Insight..

[10] Gudbjartsson DF (2007). Variants conferring risk of atrial fibrillation on chromosome 4q25. Nature.

[11] Roselli C (2018). Multi-ethnic genome-wide association study for atrial fibrillation. Nat. Genet..

[12] Hodgson J (2020). Characterization of GDF2 mutations and levels of BMP9 and BMP10 in pulmonary arterial hypertension. Am. J. Respir. Crit. Care Med..

[13] Chua W (2021). Dynamic changes of cardiovascular biomarkers after ablation for atrial fibrillation: Observations from AXAFA-AFNET5. Eur. Heart J..

[14] Mitrofan CG (2017). Bone morphogenetic protein 9 (BMP9) and BMP10 enhance tumor necrosis factor-α-induced monocyte recruitment to the vascular endothelium mainly via activin receptor-like kinase 2. J. Biol. Chem..

[15] Fan J (2012). NT-proBNP, but not ANP and C-reactive protein, is predictive of paroxysmal atrial fibrillation in patients undergoing pulmonary vein isolation. J. Interv. Card. Electrophysiol..

[16] Rienstra M, Van Gelder IC, Van den Berg MP, Boomsma F, Van Veldhuisen DJ (2006). Natriuretic peptides in patients with atrial fibrillation and advanced chronic heart failure: Determinants and prognostic value of (NT-)ANP and (NT-pro)BNP. Europace.

[17] Beck-da-Silva L, de Bold A, Fraser M, Williams K, Haddad H (2004). Brain natriuretic peptide predicts successful cardioversion in patients with atrial fibrillation and maintenance of sinus rhythm. Can. J. Cardiol..

[18] Franzini M (2013). Systematic differences between BNP immunoassays: Comparison of methods using standard protocols and quality control materials. Clin. Chim. Acta.

[19] Rørth R (2020). Comparison of BNP and NT-proBNP in patients with heart failure and reduced ejection fraction. Circ. Heart Fail..

[20] Clerico A, Passino C, Franzini M, Emdin M (2015). Cardiac biomarker testing in the clinical laboratory: Where do we stand? General overview of the methodology with special emphasis on natriuretic peptides. Clin. Chim. Acta.

[21] Casaclang-Verzosa G, Gersh BJ, Tsang TS (2008). Structural and functional remodeling of the left atrium: Clinical and therapeutic implications for atrial fibrillation. J. Am. Coll. Cardiol..

[22] Zografos T, Maniotis C, Katsivas A, Katritsis D (2014). Relationship between brain natriuretic peptides and recurrence of atrial fibrillation after successful direct current cardioversion: A meta-analysis. Pacing Clin. Electrophysiol..

[23] Kallergis EM (2010). Effect of sinus rhythm restoration after electrical cardioversion on apelin and brain natriuretic peptide prohormone levels in patients with persistent atrial fibrillation. Am. J. Cardiol..

[24] Raman K (2016). Whole blood gene expression differentiates between atrial fibrillation and sinus rhythm after cardioversion. PLoS ONE.

[25] Dong Q (2019). FGF23 regulates atrial fibrosis in atrial fibrillation by mediating the STAT3 and SMAD3 pathways. J. Cell Physiol..

[26] Faul C (2011). FGF23 induces left ventricular hypertrophy. J. Clin. Investig..

[27] Mirza MA, Larsson A, Lind L, Larsson TE (2009). Circulating fibroblast growth factor-23 is associated with vascular dysfunction in the community. Atherosclerosis.

[28] Kuga K (2020). Fibrosis growth factor 23 is a promoting factor for cardiac fibrosis in the presence of transforming growth factor-β1. PLoS ONE.

[29] Hindricks G (2020). 2020 ESC Guidelines for the diagnosis and management of atrial fibrillation developed in collaboration with the European Association of Cardio-Thoracic Surgery (EACTS). Eur. Heart J..

[30] Uittenbogaart SB (2020). Opportunistic screening versus usual care for detection of atrial fibrillation in primary care: Cluster randomised controlled trial. BMJ.

[31] Kaasenbrood F (2020). Opportunistic screening versus usual care for diagnosing atrial fibrillation in general practice: A cluster randomised controlled trial. Br. J. Gen. Pract..

[32] Schnabel RB (2014). Multiple biomarkers and atrial fibrillation in the general population. PLoS ONE.

[33] Sinner MF (2014). B-type natriuretic peptide and C-reactive protein in the prediction of atrial fibrillation risk: The CHARGE-AF Consortium of community-based cohort studies. Europace.

[34] Engdahl J (2017). Stepwise mass screening for atrial fibrillation using N-terminal pro b-type natriuretic peptide: The STROKESTOP II study design. Europace.

[35] Mihm MJ (2001). Impaired myofibrillar energetics and oxidative injury during human atrial fibrillation. Circulation.

[36] Ziberna L, Martelanc M, Franko M, Passamonti S (2016). Bilirubin is an endogenous antioxidant in human vascular endothelial cells. Sci. Rep..

[37] Jansen T (2010). Conversion of biliverdin to bilirubin by biliverdin reductase contributes to endothelial cell protection by heme oxygenase-1-evidence for direct and indirect antioxidant actions of bilirubin. J. Mol. Cell Cardiol..

[38] Hijazi Z (2014). High-sensitivity troponin T and risk stratification in patients with atrial fibrillation during treatment with apixaban or warfarin. J. Am. Coll. Cardiol..

[39] Turer AT (2011). Myocardial ischemia induced by rapid atrial pacing causes troponin T release detectable by a highly sensitive assay: Insights from a coronary sinus sampling study. J. Am. Coll. Cardiol..

[40] Ulimoen SR (2014). Improved rate control reduces cardiac troponin T levels in permanent atrial fibrillation. Clin. Cardiol..

[41] Kotecha D, Mohamed M, Shantsila E, Popescu BA, Steeds RP (2017). Is echocardiography valid and reproducible in patients with atrial fibrillation? A systematic review. Europace.

[42] Calkins H (2017). 2017 HRS/EHRA/ECAS/APHRS/SOLAECE expert consensus statement on catheter and surgical ablation of atrial fibrillation. Heart Rhythm.

