# Supplementary material for: Biomarkers associated with rhythm status after cardioversion in patients with atrial fibrillation
Source: Sci Rep. 2022 Jan 31;12:1680. doi: 10.1038/s41598-022-05769-9 (PMC8803959; doi:10.1038/s41598-022-05769-9)
Supplement: Supplementary file 1 — Supplementary Information. [file 41598_2022_5769_MOESM1_ESM.docx]

**Supporting information**

Biomarkers associated with rhythm status after cardioversion in patients with atrial fibrillation

List of 11 new biomarkers analyzed in baseline and follow-up samples

**S1 Table.** Medications of patients with and without AF recurrence before and after cardioversion

**S2 Table.** Absolute values of clinical variables at baseline and follow-up stratified by rhythm status at follow-up

**S3 Table.** Spearman correlations between BMP10 and BNP biomarkers levels at baseline

**S4 Table.** Spearman correlations between BMP10 and BNP biomarkers levels at follow-up

**S5 Table.** Models for association between changes in clinical measures and significant changes in biomarkers with rhythm status after cardioversion

**S6 Table.** Relationship between categories of percent change in BMP10 and NT-proBNP and rhythm status at follow-up

**S7 Table.** High sensitivity troponin levels at baseline and follow-up

**S1 Fig.** Box plots of percent changes in biomarker levels by rhythm status after cardioversion

**S2 Fig.** Correlation between BMP10 and BNP biomarkers levels at baseline.

Red solid lines represent the fitted mean values of BMP10 compared with BNP biomarkers.

**S3 Fig.** Correlation between BMP10 and BNP biomarkers levels at follow-up.

Red solid lines represent the fitted mean values of BMP10 compared with BNP biomarkers.

**S4 Fig.** Flow diagram of patient selection

**List of 11 new biomarkers analyzed in baseline and follow-up samples**

| **Biomarker** | **Unit** | **Description** | **Material** | **LDL**  Lower detection limit | **LLoQ**  Lower Level of Quantification | **LoB**  Limit of Blank | **LoD**  Limit of Detection | **ULoQ**  Upper Level of Quantification |
| --- | --- | --- | --- | --- | --- | --- | --- | --- |
| FABP3 | ng/mL | Fatty-acid-binding protein | Plasma | n.a. | 1.0 | - | - | 239 |
| ESM1 | ng/mL | Endothelial cell-specific molecule-1 | Plasma | 0.001 | 0.003 | - | - | 20 |
| BMP10 |  | Bone morphogenetic protein 10 | Plasma | 0.003 | 0.0 | - | - | 6.75 |
| DKK3 | ng/mL | Dickkopf-related protein 3 | Plasma | 0.003 | 0.025 | - | - | 150 |
| FGF23 | pg/mL | Fibroblast growth factor 23 | Plasma | n.a. | 4.0 | - | - | 12700 |
| IGFBP7 | ng/mL | Insulin-like growth factor-binding protein 7 | Plasma | 0.01 | 0.40 | - | - | 417 |
| MyBPC3 | pg/mL | Myosin-binding-protein C-3 | Plasma | 0.5 | 2.1 | - | - | 14550 |
| NT-proBNP | pg/mL | N-terminal pro-B-type natriuretic peptide | Plasma | 5 | 50 | 3 | 5 | 35000 |
| total NT-proBNP | pg/mL | total N-terminal pro-B-type natriuretic peptide | Plasma | 6.5 | 8.3 | - | - | 35000 |
| BNP |  | Brain natriuretic peptide | Plasma |  |  |  |  |  |
| hsTNT | pg/mL | High senstitivity troponin T | Plasma | - | 13.00 | 3.0 | 5.0 | 10000 |

**S1 Table.** Medications of patients with and without AF recurrence before and after cardioversion

|  | **Patients with AF recurrence (N=28)** | | | **Patients with sinus rhythm (N=72)** | | |
| --- | --- | --- | --- | --- | --- | --- |
| **Characteristic** | **Pre-cardioversion** | **Post-cardioversion** | **P value** | **Pre-cardioverison** | **Post-cardioverison** | **P value** |
| Medications, No. (%) |  |  |  |  |  |  |
| ß-Blocker | 18 (64) | 17 (65) | 0.99 | 58 (82) | 53 (76) | 0.29 |
| Calcium channel blockers | 5 (18) | 5 (18) | 0.99 | 10 (14) | 8 (11) | 0.50 |
| Flecainide | 1 (4) | 1 (4) | 0.99 | 3 (4) | 3 (4) | 0.99 |
| Amiodarone | 10 (36) | 10 (36) | 0.99 | 31 (44) | 27 (39) | 0.34 |
| Dronedarone | 3 (11) | 2 (8) | 0.99 | 4 (6) | 5 (7) | 0.99 |
| Digoxine | 3 (11) | 0 (0) | 0.25 | 5 (7) | 3 (4) | 0.50 |
| P value compares patients pre- vs post-cardioversion measures according to rhythm status at follow-up and are obtained from Wilcoxon matched-pairs signed-ranks test and McNemar test. | | | | | | |

**S2 Table. Absolute values of clinical variables at baseline and follow-up stratified by rhythm status at follow-up**

|  | **Baseline** | | | **Follow-up** | | |
| --- | --- | --- | --- | --- | --- | --- |
|  | **AF recurrence**  **(N=28)** | **Sinus rhythm**  **(N=72)** | **P value** | **AF recurrence**  **(N=28)** | **Sinus rhythm**  **(N=72)** | **P value** |
| **Blood pressure measures** |  |  |  |  |  |  |
| Systolic, mmHg | 137±20 | 135±17 | 0.81 | 135±20 | 134±19 | 0.99 |
| Diastolic, mmHg | 88±11 | 85±13 | 0.48 | 85±12 | 77±9 | 0.0046 |
| **Holter ECG parameters** |  |  |  |  |  |  |
| Average heart rate, /min | 79±15 | 80±17 | 0.76 | 74±22 | 59±9 | <0.001 |
| Max. heart rate, /min | 154±27 | 161±35 | 0.49 | 157±32 | 120±24 | <0.001 |
| Min. heart rate, /min | 46±12 | 44±9 | 0.79 | 47±10 | 41±7 | 0.0135 |
| P value compares laboratory and clinical values between patient with AF recurrence and patients with sinus rhythm and are calculated using Wilcoxon rank-sum test. | | | | | | |

**S3 Table. Spearman correlations between BMP10 and BNP biomarkers levels at baseline**

| **Biomarkers** | BMP10 | NT-proBNP | total NT-proBNP | BNP | Total bilirubin |
| --- | --- | --- | --- | --- | --- |
| BMP10 | 1.0000 |  |  |  |  |
| P value | - |  |  |  |  |
| NT-proBNP | 0.5220 | 1.0000 |  |  |  |
| P value | <0.001 | - |  |  |  |
| total NT-proBNP | 0.5501 | 0.9364 | 1.0000 |  |  |
| P value | <0.001 | <0.001 | - |  |  |
| BNP | 0.4361 | 0.7863 | 0.8857 | 1.0000 |  |
| P value | <0.001 | <0.001 | <0.001 | - |  |
| Total bilirubin | -0.0226 | -0.0646 | -0.0646 | 0.0033 | 1.0000 |
| P value | 0.8357 | 0.5217 | 0.5525 | 0.9757 | - |
| Values are Spearman’s rho correlations and corresponding P values. | | | | | |

**S4 Table. Spearman correlations between BMP10 and BNP biomarkers levels at follow-up**

| **Biomarkers** | BMP10 | NT-proBNP | total NT-proBNP | BNP | Total bilirubin |
| --- | --- | --- | --- | --- | --- |
| BMP10 | 1.0000 |  |  |  |  |
| P value | - |  |  |  |  |
| NT-proBNP | 0.6094 | 1.0000 |  |  |  |
| P value | <0.001 | - |  |  |  |
| total NT-proBNP | 0.6496 | 0.9181 | 1.0000 |  |  |
| P value | <0.001 | <0.001 | - |  |  |
| BNP | 0.6079 | 0.8167 | 0.9044 | 1.0000 |  |
| P value | <0.001 | <0.001 | <0.001 | - |  |
| Total bilirubin | -0.0023 | -0.0389 | -0.0069 | 0.0253 | 1.0000 |
| P value | 0.9827 | 0.7191 | 0.9492 | 0.8148 | - |
| Values are Spearman’s rho correlations and corresponding P values. | | | | | |

**S5 Table. Models for association between changes in clinical measures and significant changes in biomarkers with rhythm status after cardioversion**

|  | **Predictor** | | | | | | | | |
| --- | --- | --- | --- | --- | --- | --- | --- | --- | --- |
|  | **Change in BMP10, ng/mL** | | | **Change in NT-proBNP, pg/mL** | | | **Change in total bilirubin, umol/L** | | |
| **Model** | **β (95% CI)** | **Standardized β (95% CI)*** | **P value** | **β (95% CI)** | **Standardized β**  **(95% CI)** | **P value** | **β (95% CI)** | **Standardized β**  **(95% CI)** | **P value** |
| Adjusted for change in diastolic BP, mmHg | -4.30  (-6.71 to -1.88) | -2.06  (-3.22 to -0.90) | 0.001 | -0.007  (-0.01 to -0.003) | -5.63  (-8.42 to -2.83) | <0.001 | -0.25  (-0.49 to -0.01) | -0.91  (-1.77 to -0.05) | 0.038 |
| Adjusted for change in average heart rate, /min | -4.47  (-7.00 to -1.93) | -2.09  (-3.28 to -0.91) | 0.001 | -0.006  (-0.009 to -0.003) | -3.53  (-5.33 to -1.73) | <0.001 | -0.27  (-0.51 to -0.02) | -0.94  (-1.81 to -0.07) | 0.034 |
| Adjusted for change in max. heart rate, /min | -5.95  (-9.29 to -2.71) | -2.79  (-4.31 to -1.27) | <0.001 | -0.006  (-0.009 to -0.003) | -3.54  (-5.49 to -1.58) | <0.001 | -0.23  (-0.51 to 0.05) | -0.82  (-1.82 to 0.18) | 0.11 |
| Abbreviations: BP, blood pressure.  Estimates were obtained from logistic regression models where rhythm status at follow-up was the dependent variable and change of biomarker was the independent variable. Covariates were selected based on the significance level of <0.01. All models were adjusted for biomarker levels at baseline and significant changes in clinical measures.  * Standardized β coefficients are defined as per 1-SD decrease in biomarker change. | | | | | | | | | |

**S6 Table. Relationship between categories of percent change in BMP10 and NT-proBNP and rhythm status at follow-up**

|  | **Change BMP10 below median, change** **NT-proBNP below median** | **Change BMP10 below median, change NT-proBNP above median** | **Change BMP10 above median, change NT-proBNP below median** | **Change BMP10 above median, change NT-proBNP above median** | **P value** | |
| --- | --- | --- | --- | --- | --- | --- |
|  |  |  |  |  | Unadjusted | Adjusted^a^ |
| **No./No. (%)*** | 0/33 (0) | 6/17 (35) | 2/17 (12) | 19/32 (59) | <0.001 | <0.001 |
| ^*^ Number of patients with AF recurrence/total number of patients.  ^a^ P value is from logistic regression models and adjusted for age (years), sex, BMI, smoking status (active, past, never), hypertension, heart failure. | | | | | | |

**S7 Table. Baseline characteristics of patients according to high sensitivity troponin T levels**

| **Characteristic** | **Patients with hsTnT levels >14 pg/mL at baseline and follow-up (N=28)** | **Patients with hsTnT levels <14 pg/mL at baseline and follow-up (N=72)** | **P value** |
| --- | --- | --- | --- |
| Age, years | 69±6 | 64±9 | 0.05 |
| Sex, No. (%) |  |  | 0.010 |
| Women | 2 (7) | 23 (32) |  |
| Men | 26 (93) | 49 (68) |  |
| Body-mass index, median (IQR), kg/m^2^ | 25.0 (24.0-28.6) | 27.2 (24.6-29.6) | 0.21 |
| Smoking status, No. (%) |  |  | 0.99 |
| Active | 3 (11) | 8 (11) |  |
| Past | 14 (50) | 35 (49) |  |
| Never | 11 (39) | 29 (40) |  |
| Months since atrial fibrillation diagnosis,  median (IQR) | 11 (2-56) | 19 (4-78) | 0.32 |
| Medical history, No. (%) |  |  |  |
| Hypertension | 18 (64) | 46 (64) | 0.99 |
| Diabetes mellitus | 1 (4) | 5 (7) | 0.99 |
| Stroke or transient ischemic attack | 3 (11) | 8 (11) | 0.99 |
| Myocardial infarction | 4 (14) | 4 (6) | 0.22 |
| Percutaneous coronary intervention | 4 (14) | 8 (11) | 0.74 |
| Heart failure | 7 (25) | 7 (10) | 0.05 |
| Medication, No. (%) |  |  |  |
| Beta-blocker | 24 (86) | 52 (73) | 0.29 |
| Calcium channel blockers | 3 (11) | 12 (17) | 0.55 |
| Flecainide | 0 (0) | 4 (6) | 0.58 |
| Amiodarone | 14 (50) | 27 (38) | 0.37 |
| Dronedarone | 0 (0) | 7 (10) | 0.19 |
| Digoxin or digitoxin | 2 (7) | 1 (1) | 0.22 |
| AF recurrence at follow-up, No. (%) | 10 (36) | 18 (25) | 0.33 |
| Values are means±SD.or median (IQR) and numbers (percent).  P values compare patients between with AF recurrence and sinus rhythm and are obtained from Wilcoxon rank-sum tests for continuous variables and Fisher’s exact tests for categorical variables. | | | |

**S1 Fig. Box plots of percent changes in biomarker levels by rhythm status after cardioversion**

**S2 Fig. Correlation between BMP10 and BNP and bilirubin levels at baseline**

**S3 Fig. Correlation between BMP10 and BNP and bilirubin levels at follow-up**

**S4 Fig. Flow diagram of patient selection**
